# Optical Coherence Tomography Angiography Findings in a Case of Cosmetic Laser Induced Retinal Injury

**DOI:** 10.7759/cureus.5760

**Published:** 2019-09-25

**Authors:** Fabliha Anbar, Matthew Flood, Saad Shaikh

**Affiliations:** 1 Medical Education and Simulation, University of Central Florida College of Medicine, Orlando, USA; 2 Ophthalmology, Stillwater Eyecare, Stillwater, USA; 3 Ophthalmology, University of Central Florida College of Medicine, Orlando, USA

**Keywords:** choroid, choriocapillaris, iris, laser injury, melanin, photocoagulation, retinal pigment epithelial, alexandrite laser

## Abstract

We report the first known case of iris and retinal injury using optical coherence tomography angiography (OCTA) images following cosmetic laser injury. A 23-year-old female developed left iris and retinal injury post inadvertent firing of a 755 nm Alexandrite cosmetic laser. The patient had no significant past medical history and the injury resulted from inappropriate eye protection during laser use. Injury from the laser caused damage to the retinal pigment epithelium (RPE), iris pigment epithelium (IPE), and ciliary body epithelium (CPE). The OCTA imaging modality detected the vascular injury caused by the Alexandrite 755 nm laser to the choroid and RPE with subsequent images visualizing the healing response in the months postinjury.

## Introduction

The popularity of cosmetic laser procedures results in a rise in ocular injuries with the most reported injury occurring from laser hair removal of the periorbital areas. According to Huang et al. from 1985 to 2012, laser hair removal was the most commonly litigated procedure, as well as one that caused detrimental visual and cosmetic side effects to patients and medical technicians alike [1]. Herein we present a case of laser injury that developed immediately after inadvertent firing of an Alexandrite 755 nm laser.

## Case presentation

A 23-year-old female with no significant past medical history was referred to the ophthalmology clinic after sustaining a cosmetic laser injury to her left eye. The patient works as a medical assistant at a dermatology practice where she performs laser hair removal with a Candela GentleLase Mini laser (755 nm Alexandrite). She was not wearing protective glasses when the laser inadvertently fired. She reported immediate pain with light sensitivity. Physical exam findings postinjury showed pinhole to 20/30-2 vision in both eyes, with intraocular pressure of 14 in the right eye and 12 in the left eye. The patient’s left eye had an irregular pupil and was reactive from 4 to 3 mm. Her right pupil was normal and reactive from 5 to 3 mm. No afferent pupillary defect was noted, and her motility and confrontational fields remained full.

**Figure 1:**
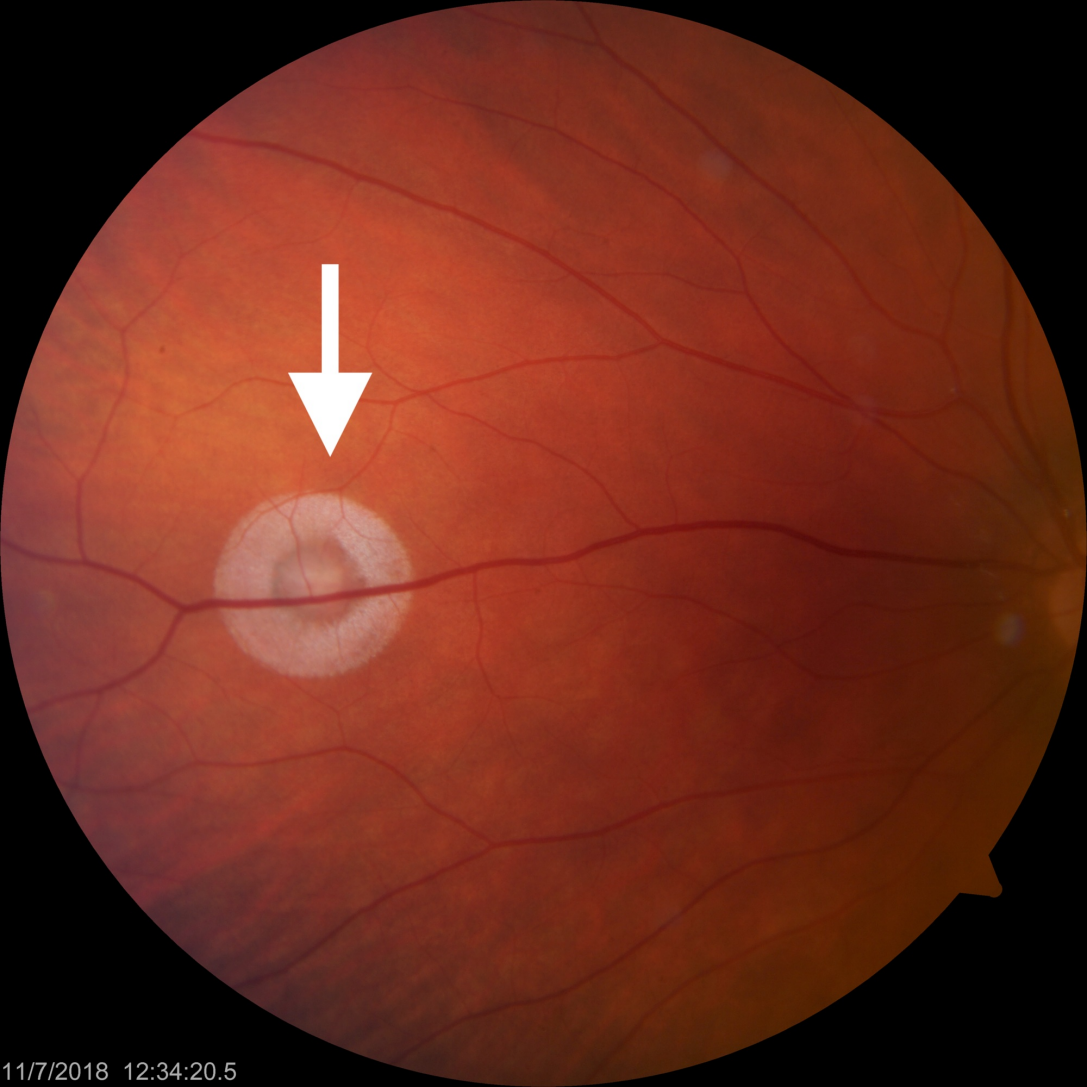
Fundus photo of the left eye. White lesion corresponding to site of acute laser injury in the nasal periphery, centrally intense with collateral spread (white arrow).

Slit lamp examination revealed irregularity of her left pupil with temporal and nasal transillumination defects in the left iris. The pupillary border was frayed in the temporal region, and there were 4+ pigmented cells in the anterior chamber of her left eye. The lens was clear. On dilated fundus examination, a one disc diameter (DD) circular, white lesion was found in the nasal retina (Figure 1) of the left eye. The right eye did not reveal any changes.

**Figure 2:**
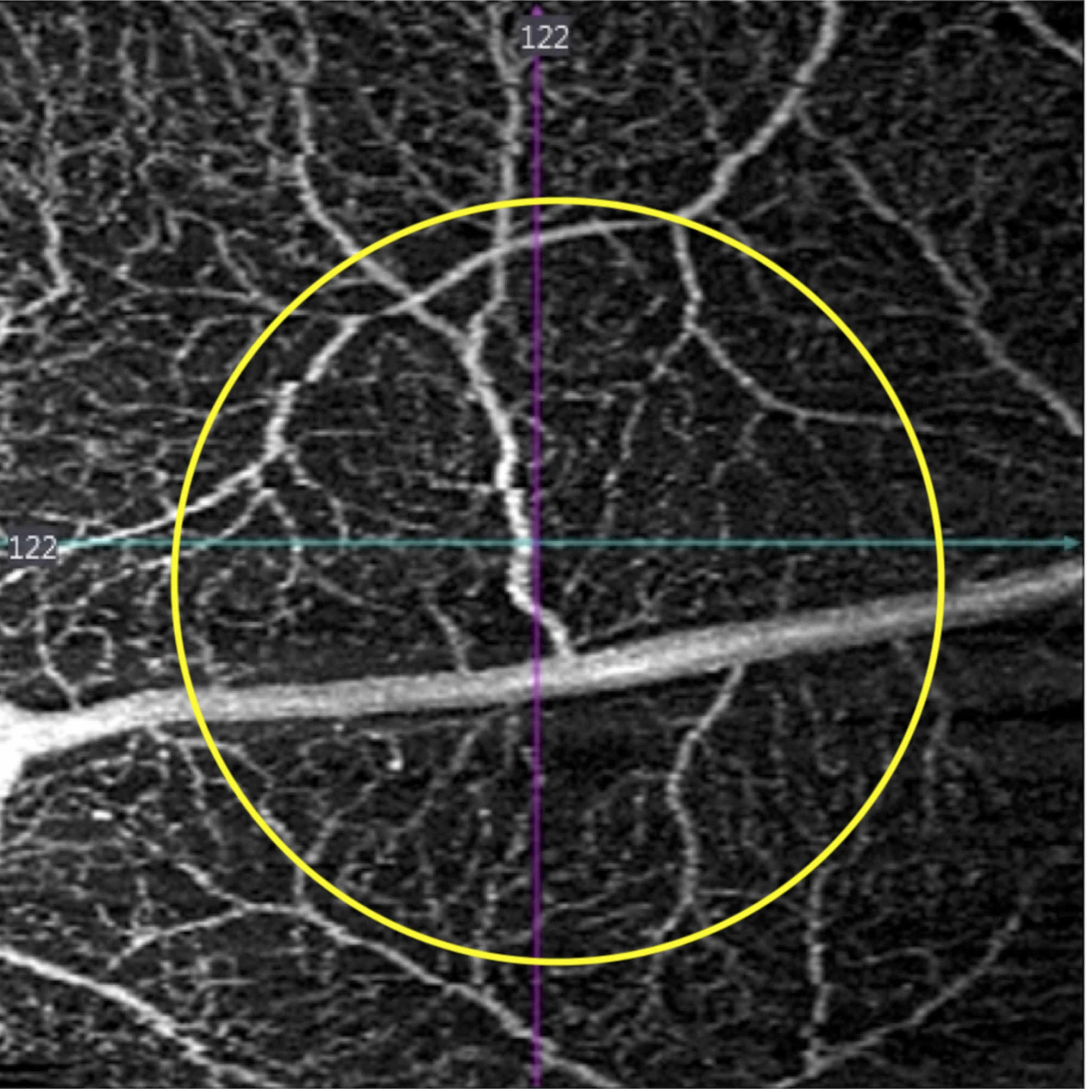
One week after injury, composite retinal vascular optical coherence tomography angiography slab demonstrates intact retinal vasculature (yellow circle delineates the margins of laser injury).

**Figure 3:**
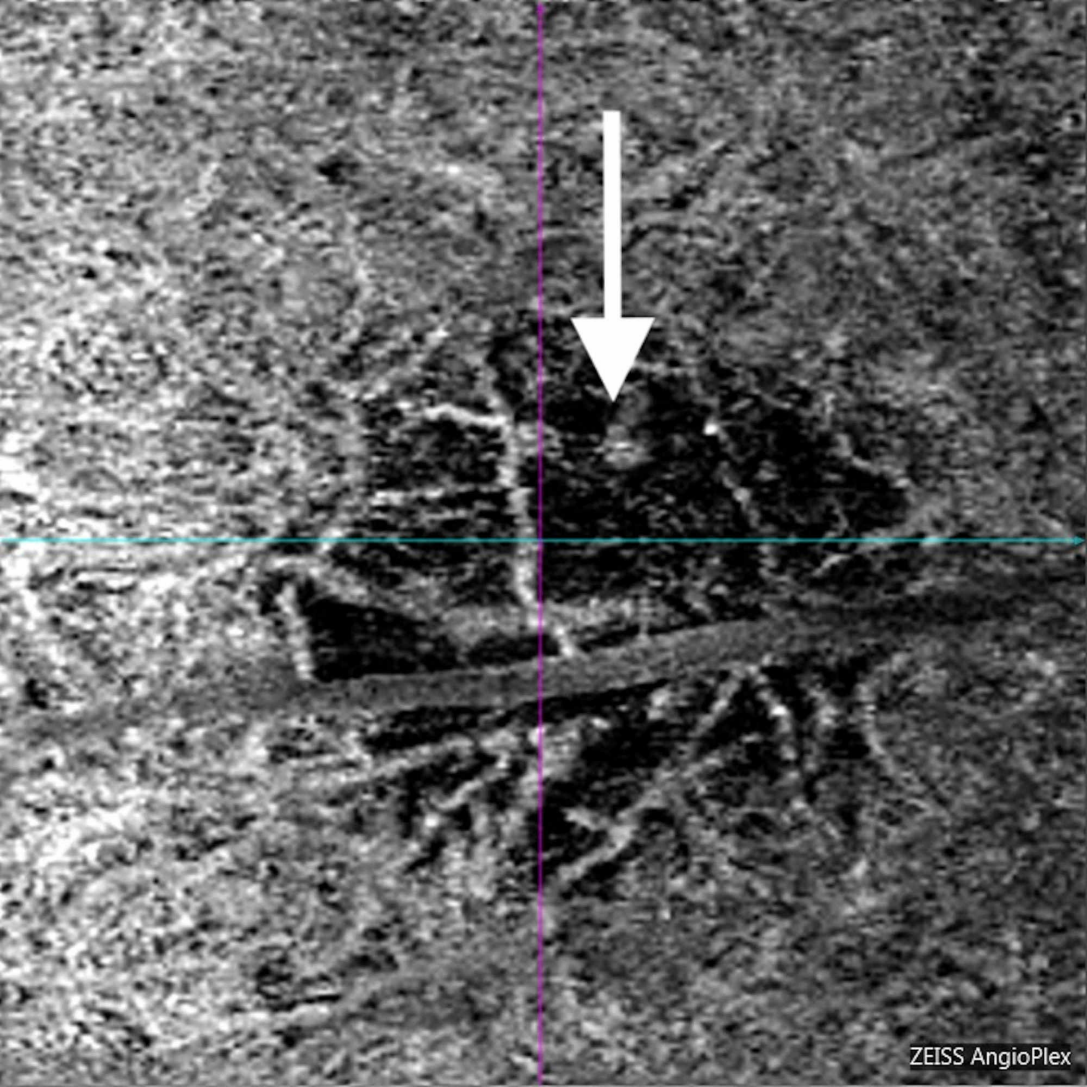
Optical coherence tomography angiogram choriocapillaris slab. Extensive focal damage with ablation of the choriocapillaris (white arrow) is noted.

**Figure 4:**
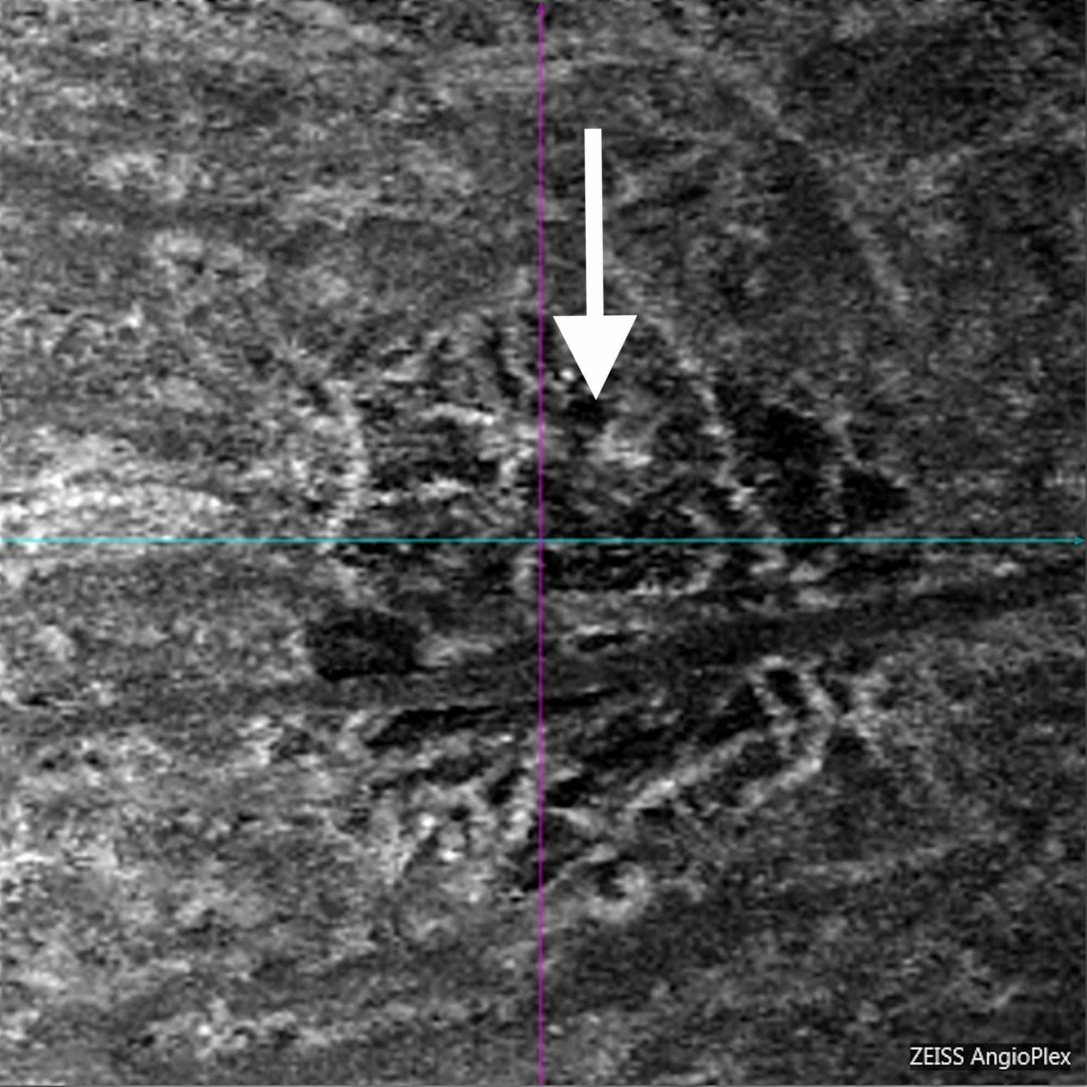
Optical coherence tomography angiogram choroidal vasculature slab. Extensive focal damage with ablation of the choroid (white arrow).

The patient was started on prednisolone acetate drops every two hours and cyclopentolate drops twice a day. At the one-week follow up visit, the patient reported improvement in pain but persistent light sensitivity. Her examination demonstrated 2+ anterior pigmented cells. The retinal lesion demonstrated central pigmentary proliferation and peripheral depigmentation. Optical coherence tomography angiography (OCTA) demonstrated no damage to the retinal vascular layers. However, extensive focal damage was noted to the choriocapillaris and choroid (Figures 2-4). In addition, the patient developed mild headaches with work requiring near vision correction. Cyclopentolate was discontinued and prednisolone acetate was decreased to four times a day.

**Figure 5:**
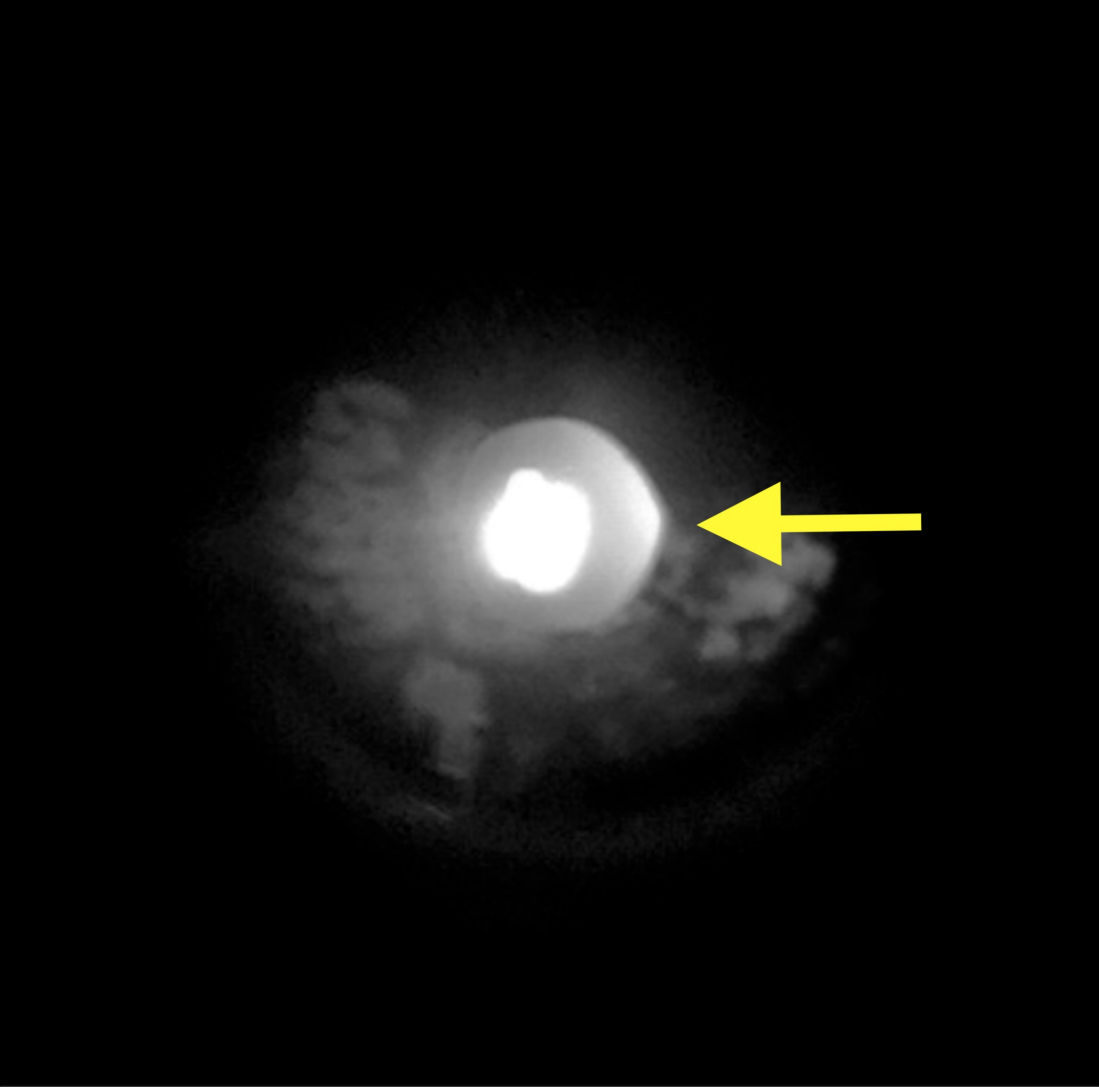
Left eye anterior segment photo demonstrating irregular pupil and extensive iris atrophy (yellow arrow).

**Figure 6:**
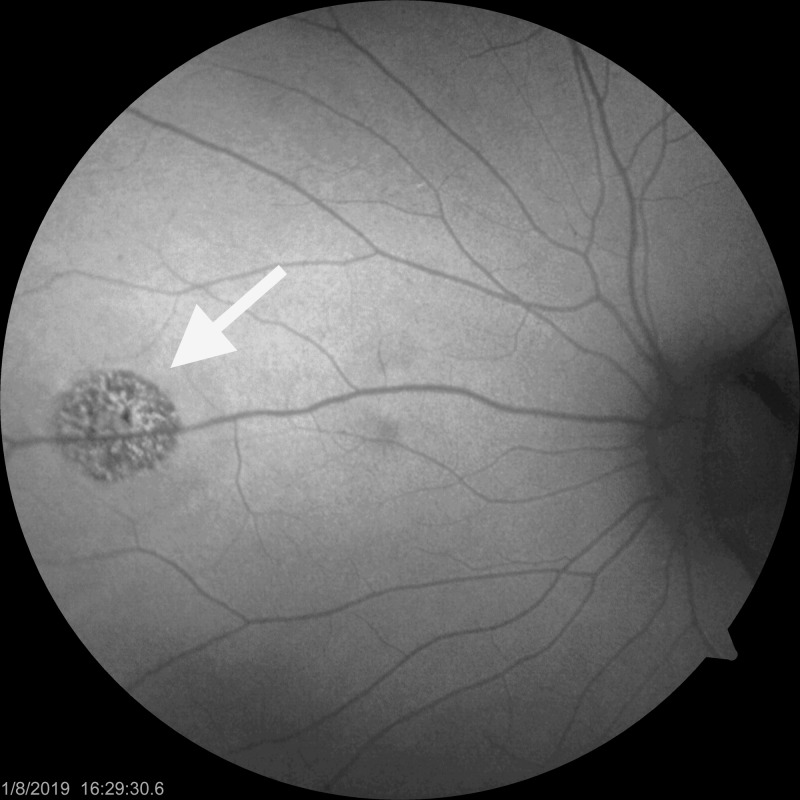
Left eye fundus photo demonstrating fully pigmented chorioretinal lesion (white arrow).

**Figure 7:**
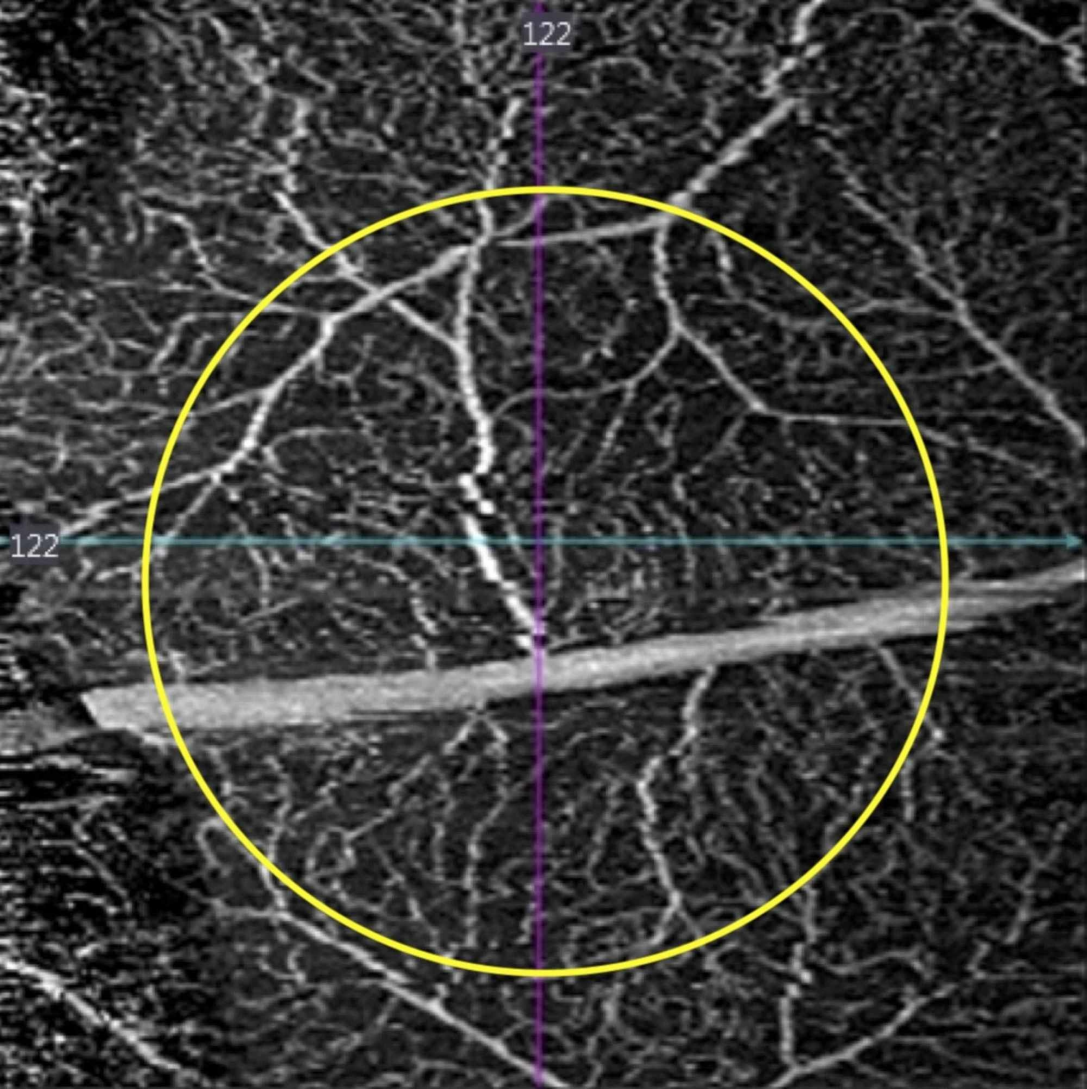
Sixty-two days after injury, composite retinal vascular optical coherence tomography angiography slab demonstrates intact retinal vasculature (yellow circle delineates the margins of laser injury).

**Figure 8:**
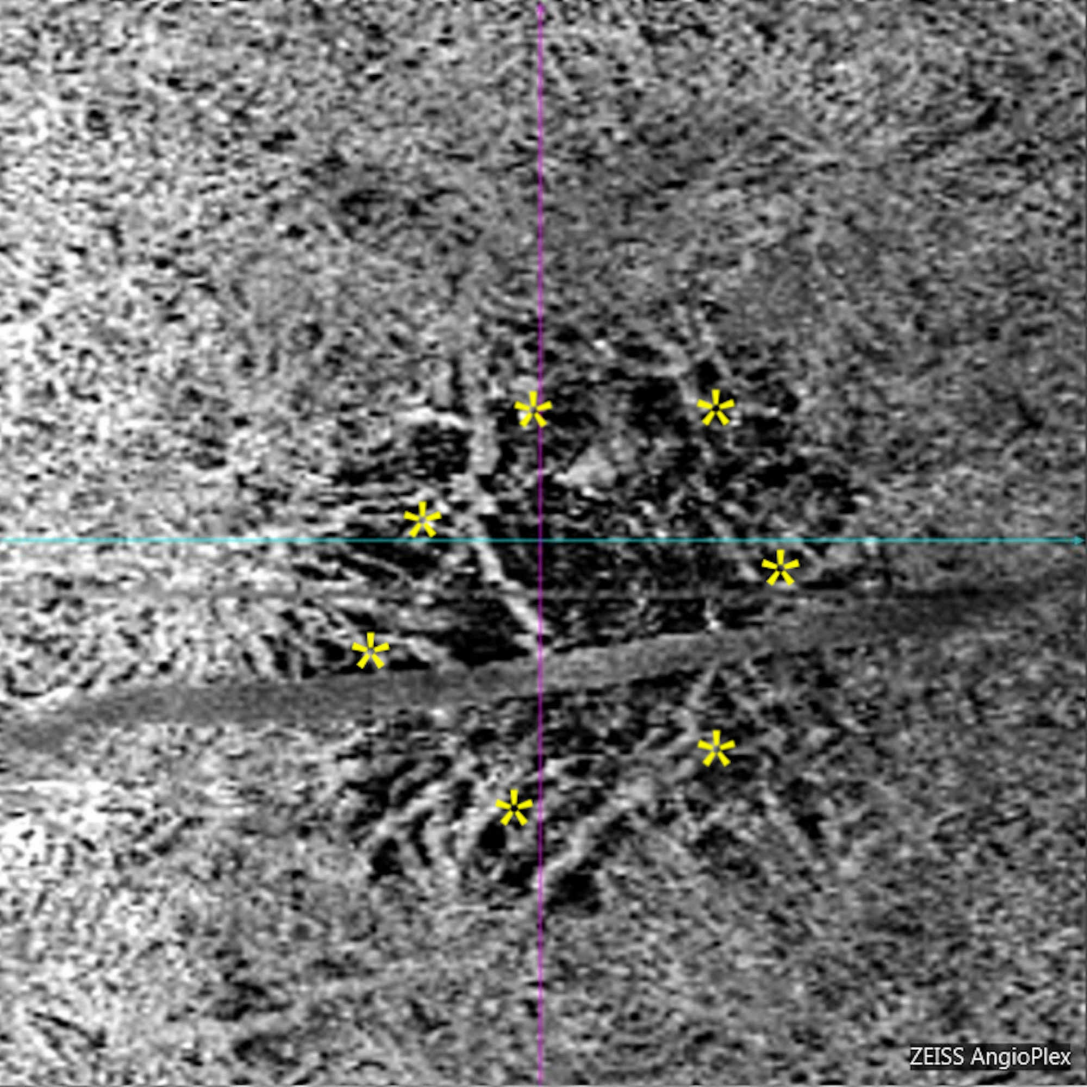
Optical coherence tomography angiogram choriocapillaris slab. The choriocapillaris has begun to revascularize from the periphery of the lesion (asterisks).

**Figure 9:**
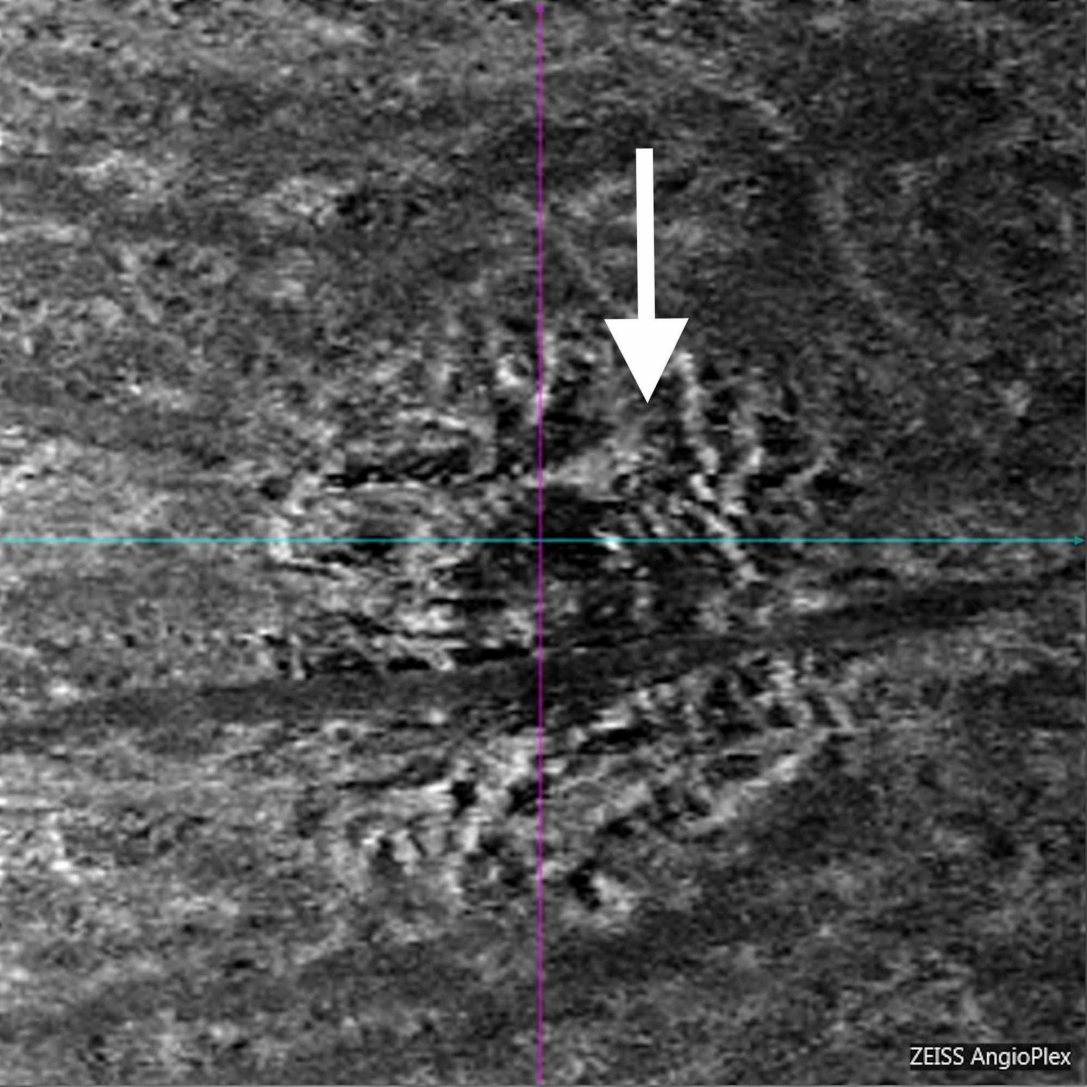
Optical coherence tomography angiogram choroidal vasculature slab. Extensive atrophy of choroid persists (white arrow).

At the 26-day follow-up visit, the patient had continued light sensitivity and 2+ anterior pigmented cells. The patient was asked to taper prednisolone use. Follow-up exam at 62-days postinjury showed complete resolution of the anterior segment cellular reaction and photophobia. A mildly irregular pupil was noted with extensive iris atrophy (Figure 5). The retinal lesion was fully pigmented (Figure 6). OCTA demonstrated intact retinal vascular layers and although focal atrophy of the choroidal vessels persisted, the choriocapillaris layer had begun to repopulate from the outer margins (Figures 7-9). The patient complained of white washed vision and was concerned about the cosmesis of her iris to which a recommendation to visit an optometrist for colored contact lenses was made.

## Discussion

As in our reported case, the 755 nm Alexandrite laser is most often used for laser hair removal and has the most reported laser-caused ocular complications [2]. Lasers within this wavelength work on the basis of selective photothermolysis and selectively target melanin. Ocular melanin is found in the uveal tract which includes the iridal, ciliary, and choroidal melanocytes, in addition to the pigment epithelial layers of the retina, iris, and ciliary bodies [3]. Absorption of the waves in these layers leads to damage with side effects coinciding with the location of injury [4].

This case is the first known report of OCTA imaging used to demonstrate cosmetic laser injury to the retina and choroid. Based on the patient’s history, it is presumed she set the cosmetic laser to her left and it fired into her left eye as she looked straight ahead. This resulted in direct injury to her iris and retina. The location of injury in this case conforms with the melanin regions of the eye.

The use of OCTA images has the significant advantage over fluorescein and indocyanine green angiography of allowing sublayer en face visualization [5-6]. The OCTA images in our report visualized the intact retinal vessels and simultaneous damage to choriocapillaris and choroid from initial presentation to one-month follow-up. Of note, it appeared that reperfusion of the choriocapillaris from the retinal periphery had begun at final follow-up. Although neither OCTA nor angiography is required for the diagnosis of injury in this case, the former, at comparatively lower cost and noninvasively, can add to our understanding of the extent of injury to the vascular layers of the retina and choroid. 

## Conclusions

The increased prevalence of laser use for cosmetic procedures such as hair removal and decreasing pigmentation of the skin has conformed to a consequent rise in ocular injuries. A majority of reported cases were due to laser hair treatment with inadequate eye protection or Bell’s phenomenon. While injuries to the patient are more common, in our case we present ocular injury to the medical assistant due to inadvertent laser firing and inadequate ocular protection. Ultimately, the patient and medical assistant performing the procedure are both at risk of laser injury. With the FDA approval of at-home laser hair removal devices, the use of adequate eye protection and advanced trained personnel in performing procedures near the eye is essential. In addition, ophthalmologists need to be cognizant of this type of injury in order to provide optimal care.
